# Time to Grow: Circadian Clock Controls Plant Hormone Signaling and Response

**DOI:** 10.1371/journal.pbio.0050227

**Published:** 2007-08-07

**Authors:** Mary Hoff

Unable to move from one place to another in response to changing conditions, plants survive by moderating their growth to make the most of their circumstances. Plants sprout new leaves and roots and grow toward or away from light and gravity, all of which requires the growth hormone auxin.

Buried in historical scientific literature are hints that auxin might affect plants differently at different times of day. This makes sense from an adaptive standpoint: an internal clock allows plants to respond to stimuli such as sun, rain, and being eaten in the context of regular rhythms of the inescapable world around them. But how do they do it? The mechanisms tuning growth to internal rhythms, with implications for everything from growing crops to protecting biodiversity in the face of global climate change, have remained a mystery. Now, Michael Covington and Stacey Harmer have discovered clues to the roots of rhythmic growth.

The researchers began by identifying genes in *Arabidopsis*, the botanical equivalent of the laboratory rat, showing a circadian (daily) rhythm in transcription as indicated by changes in messenger RNA abundance. A surprising number of these genes are involved in auxin signaling.

But at what stage? Looking closely at the genes involved, the researchers determined that auxin and the circadian clock are intimately intertwined virtually every step of the way. Genes regulating the production of auxin are clock controlled. So are genes that make auxin carriers, that inactivate auxin, and that make the receptor molecules that sense auxin and subsequently alter the transcription of other genes. Many of the genes auxin induces also show a circadian rhythm.

To explore in more detail where the circadian clock and auxin signaling pathways intersect, the researchers used a luciferase reporter assay—basically, a firefly gene inserted into the genome that produces a glow when it and associated genes are activated.

Covington and Harmer began by determining whether auxin affects circadian rhythms. Using the luciferase reporter assay to determine when genes controlling circadian rhythm were activated, they found that in the short term, added auxin didn’t affect the phase of the rhythm, but at some concentrations it increased period length and reduced amplitude (that is, the magnitude of fluctuation in the apparent activity of reporter genes). Prolonged auxin treatment slightly lengthened the period and dramatically reduced the amplitude of the rhythm.

To determine whether rhythms affect auxin’s effects on gene transcription, the researchers inserted the firefly gene into plants in a way that caused them to glow when an auxin promoter, eDR5, is activated. These “eDR5::LUC” plants showed a circadian rhythm in reporter gene expression, indicating that auxin signaling is indeed clock controlled.

**Figure:**
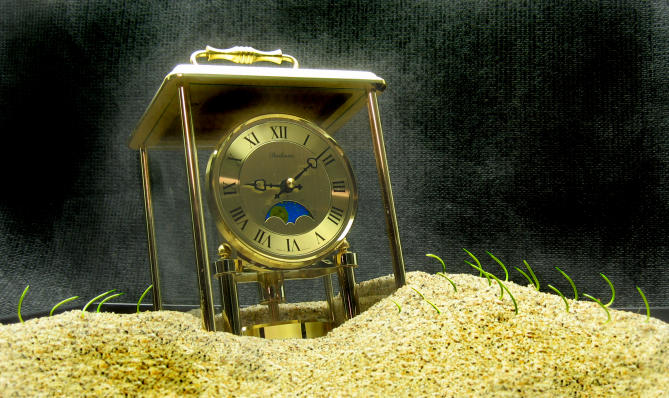
The circadian regulation of auxin responses suggests that many aspects of plant physiology not previously thought to be under circadian control may show time-of-day-specific sensitivity. (Image: Michael F. Covington and Kimberly J. Cope)

Next, the researchers explored whether the circadian fluctuation of auxin levels is what causes the rhythm in the transcription of auxin-related genes by administering auxin to eDR5::LUC plants. The plants responded with a quick glow, but not with change in phase or amplitude of glowing over the long term. The researchers then repeated the same experiment using plants in which auxin transport mechanisms were disrupted, with similar results. Their conclusion: the rhythms in transcription of auxin-controlled genes are not due to rhythms in auxin synthesis or transport.

Circadian clocks have been found to alter responses to certain stimuli, a phenomenon known as “gating.” To find out whether the sensitivity of plants to exogenous auxin is altered by their biological clock, the researchers administered auxin to eDR5::LUC seedlings under constant light conditions. When auxin was given during day, the plants showed little if any increase in glowing; when it was given at night, they showed a clear response that peaked just before dawn. This differential response affirmed that auxin signaling is indeed gated by the plant’s internal rhythms.

To determine whether gating shows up in growth patterns as well as in transcription, the researchers observed the growth of the embryonic shoot under continuous light in both control and auxin-treated seedlings. Control plants showed a rhythm in elongation. Auxin treatment boosted growth when given during what would have been night, but not during what would have been day—the same rhythm shown previously in transcription activation. The researchers concluded that growth is gated by auxin, too.

Buoyed by their findings, the researchers look forward to exploring the interplay of rhythms and auxin action with respect to various aspects of plant development, including photo- and gravitropism and the development of specialized structures. Such studies will provide additional valuable insights into how circadian rhythms help plants adapt to the world around them.

